# Effects of Aphid Density and Plant Taxa on Predatory Ladybeetle Abundance at Field and Landscape Scales

**DOI:** 10.3390/insects11100695

**Published:** 2020-10-13

**Authors:** Hongsheng Pan, Bing Liu, Coline C. Jaworski, Long Yang, Yongqiang Liu, Nicolas Desneux, Eva Thomine, Yanhui Lu

**Affiliations:** 1 State Key Laboratory for Biology of Plant Diseases and Insect Pests, Institute of Plant Protection, Chinese Academy of Agricultural Sciences, Beijing 100193, China; panhongsheng0715@163.com (H.P.); liubing1945@126.com (B.L.); yanglong9005@163.com (L.Y.); lyq364467268@163.com (Y.L.); 2 Scientific Observing and Experimental Station of Crop Pests in Korla, Ministry of Agriculture and Rural Affairs, Institute of Plant Protection, Xinjiang Academy of Agricultural Sciences, Urumqi 830091, China; 3 Department of Zoology, University of Oxford, Oxford OX1 3PS, UK; jaworskicoline@yahoo.fr; 4 Université Côte D’Azur, INRAE, CNRS, UMR ISA, 06000 Nice, France; nicolas.desneux@inra.fr (N.D.); eva.thomine@gmail.com (E.T.)

**Keywords:** habitat use, host plant shift, predator-prey interaction, plant diversity, conservation biological control, landscape heterogeneity

## Abstract

**Simple Summary:**

In agroecosystems, predatory ladybeetles play an important role in suppressing aphid populations. How ladybeetles make use of host plant diversity in multicropping landscapes has rarely been documented in China. In this study, we examined the relationship between aphid densities and ladybeetle densities at both the local field and landscape scales. Overall, we found that there was a positive correlation between aphid densities and ladybeetle densities. However, plant taxa had no significant influence on predatory ladybeetle abundance at the local field scale. In addition, the effect of aphids on ladybeetles abundance was influenced by the crop type and growing season at the regional landscape scale. There was a significant positive correlation between aphid and ladybeetle populations on cotton only in July and August, whereas the correlation was significant for maize throughout the whole growing season. The *δ*^13^C value indicated that most prey aphids for ladybeetles originated from crops where aphids are abundant (cotton in June and July; both maize and cotton in August). These findings improved our understanding of the migration and dispersal of ladybeetles among different habitats and plant species and provided insight into the promotion of regional conservation and pest control of natural enemies in Northern China.

**Abstract:**

In agroecosystems, predatory ladybeetles play an important role in restraining aphid population growth and suppressing aphid populations. They can adapt to various habitats and make use of various aphid species associated with multiple host plants during their life cycle. Agricultural landscapes in China are composed of a mosaic of small fields with a diverse range of crops, and how ladybeetles make use of host plant diversity in such landscapes has rarely been documented. In this study, we examined the relationship between aphid densities and ladybeetle densities in two different settings: (i) on the majority of plant species (including crops, trees, and weeds) at a local field scale in 2013 and 2014, and (ii) in paired cotton and maize crop fields at a regional landscape scale in 2013. Overall, we found that aphid abundance determined predatory ladybeetle abundance at both the local field and landscape scales, and there was a positive correlation between aphid densities and ladybeetle densities. However, plant taxa had no significant influence on the predatory ladybeetle abundance at the local field scale. In addition, the effect of aphids on ladybeetles abundance was influenced by the crop type and growing season at the regional landscape scale. There was a significant positive correlation between aphids and ladybeetles populations on cotton only in July and August, whereas the correlation was significant for maize throughout the whole growing season. We also conducted an analysis of the stable carbon isotope ratios of the adult ladybeetles caught in cotton and maize fields (C_3_ and C_4_ crops, respectively) in a regional landscape-scale survey in 2013. The *δ*^13^Cvalue indicated that most prey aphids for ladybeetles originated from crops where aphids are abundant (cotton in June and July; both maize and cotton in August).These findings improved our understanding of the migration and dispersal of ladybeetles among different habitats and plant species and provided insight into the promotion of the regional conservation and pest control of natural enemies in northern China.

## 1. Introduction

Both adult and larvalladybeetles are important predators of various aphid speciesin agroecosystems [1,2]. Combinations of laboratory, greenhouse, and field studies carried out in multiple agricultural systems have reported the contributions and importance of predatory ladybeetles to both decrease aphid population growth and lower peak aphid densities, making them essential biological control agents [3,4,5,6]. For example, they can cause strong, season-long suppression of aphid populations in soybean fields [7]. Many factors could affect the population occurrence of predatory ladybeetles and their potential ecosystem services through pest suppression, but prey abundance is the most important and direct component [1,8,9]. Herbivores are highly dependent on their hosts—various types of plants, such as crops, forests, grass, or weeds, or other phytogroups; plant diversity and functionality could directly affect the diversity and abundance of herbivores and usually influence the higher trophic level-predatory enemies through interactions between plant–herbivore–predator [10,11,12,13].

Most of generalist predatory ladybeetle adults and larvae rely on aphids as a large part of their diets, although they may occasionally use alternative floral resources such as plant pollen and nectar at low aphid density [14,15,16,17]. Adult ladybeetles usually disperse to various habitats and lay eggs on diversifyinghost plant species to utilize aphid preys during the same season [9], and their mobility is critical in enabling them to cope with transient and unpredictable food resources that are scattered in both space and time [9,18,19,20,21]. Aphidophagous ladybeetle adults are highly mobile but become less active and lay eggs at sites with high aphid densities to provide adequate food resources to larvae [1,22,23,24,25]. However, landscape composition and plant diversity were likely to affect their foraging behavior and capacity to locate food resources [26,27,28,29]. The quality of landscape habitat, usually determined by the availability of food resources, is a major driver for the movement of natural enemies among crops [30,31]. Compared with the monoculture landscape, the incidence for natural enemies’ access to high quality habitat appear much higher within a complex one, which is beneficial for the biocontrol services contributed by natural enemies [32,33].

Carbon isotope analyses have provided insights into ladybeetle movements across fields with different plant types. Indeed, plants using a C_3_ versus a C_4_ photosynthetic pathway have a different ratio of ^13^C over ^12^C (i.e., *δ*^13^C), and this signature is transferred to the body of insect herbivores and their own insect predators [34,35,36,37,38]. Because of their low mobility, apterous aphids’ carbon isotope signature is entirely determined by their host plant [39]. Therefore, the combined information on ladybeetle and aphid locations and on ladybeetle carbon isotope signatures makes it possible to estimate ladybeetle movement between habitats composed of C_3_ and C_4_ plants [19] and thus to obtain insights into their host plant use.

In the multicropping system of northern China, *Harmonia axyridis* Pallas, *Propylea japonica* Thunberg, and *Coccinellaseptempunctata* L. are commongeneralist predatory ladybeetle species that play a major role in suppressing pest populations, especially those of various key aphid species [5,40,41]. For example, ladybeetles can effectively delay the establishment and subsequent population growth of the cotton aphid *Aphis gossypii* Glover during the cotton growing season [42], and *P. japonica* adults can aggregate on plants with high aphid density in agricultural ecosystems composed of cotton and maize [38]. A better knowledge of the responses of ladybeetles to aphid densities and of their temporal use of different host plant species will help promote their conservation and improve biocontrol services by preserving preferred habitats and enhancing their movements between crop fields.

We hypothesized that the aphid density and plant taxa is a key determinant of the population abundanceof predatory ladybeetles in the agroecosystems. We first surveyed the relationship between aphid densities and ladybeetle densities in habitats with the majority of plant species (including crops, trees, and weeds) locally present at a local field scale in 2013 and 2014, and the survey was simultaneously conducted in paired cotton and maize fields (two important crops with overlapping growth periods in northern China) at a regional landscape scale in 2013. In addition, we measured the movement of the ladybeetles by calculating the stable carbon isotope ratios of the adult ladybeetles collected in cotton and maize fields from our regional landscape-scale survey. Our study provides insight into how aphids and plant taxa drive ladybeetle occurrence or migration in various habitats and at various spatiotemporal scales in agricultural ecosystems.

## 2. Materials and Methods

### 2.1. Relationship between Aphid and Ladybeetle Abundance at the Field Scale

We examined the correlation between aphid densities and ladybeetle abundance in a mosaic, multicropping local farmland (approx. 50 ha.) near the Langfang Experimental Station, Chinese Academy of Agricultural Sciences (CAAS) (39.53° N, 116.70° E), in Hebei Province, China, from early May to early September in 2013 and 2014. This farmland corresponded to the typical local small-scale multicropping pattern, with multiple small fields (0.2 to 0.5 ha.) of each crop. Overall, wheat was the main crop in spring and early summer, and maize was the dominant crop in summer, along with many minor vegetable crops (e.g., tomato, cucumber, Chinese cabbage), fruit trees (e.g., Chinese date, grape, peach), and timber trees (e.g., poplar). A total of 96 common plant species in the farmland were sampled in 2013 (78 species) and 2014 (80 species) (Table S1). They were classified into three plant functional groups (called “Plant_FG”), i.e., crop plants (31 species), trees (12 species), and weeds (53 species) (Table S1). Plant species were identified using regional weed guides [43] or with the expertise of CAAS plant taxonomists. Aphids and ladybeetles were counted every 10 days and, in total, 13 times each year, on 10 to 20 samples per plant species following Pan et al. [44]. Briefly, sampling consisted of visual plant inspections to count the number ofaphids, as well as adult and larval ladybeetles on the same plant. The identity of adult and larval ladybeetles collected was assessed based on morphological features [45]. For abundant herbaceous species, a sample consisted of a total area of 2 to 20 m^2^, while for scattered herbaceous species with small distributions, all plants in a patch ranging from 0.02 m^2^ to 0.50 m^2^ were sampled. For trees, 10 young branches were randomly sampled (length of 30 cm). The population densities of the aphids or ladybeetles were unified by upscaling or downscaling the sampling area (the crops and weeds were mapped to 1 m^2^, and 10 branches per tree weresampled) of each plant species on each sampling date.

### 2.2. Relationship between Aphid and Ladybeetle Abundancein Paired Cotton and Maize Fields at the Regional Landscape Scale

We assessed the effects of aphids on the occurrence of predatory ladybeetle metapopulations on multiple scales. At the regional landscape scale, we surveyed 83 study sites distributed aroundLangfang city and Xiongxian County (belonging to Baoding city) in Hebei Province and around the cities of Wuqing and Jinghai in Tianjin Province in northern China in 2013 (see Figure S1). The distance between the two study sites was averagely 3–4 km. In each study site, two adjacent crop fields were cultivated with cotton (a C_3_ plant, crop variety “GuoXin”) and maize (a C_4_ plant, crop variety “ZhengDan958”), and each had an area greater than 5000 m^2^.The aphid and ladybeetle densities were measured three times: mid-June, mid-July, and mid-August, which corresponded to the budding, flowering, and bolling stages of cotton, and the seedling, whorl, and silking stages of maize. No insecticide was sprayed during the trials or study periods (preventing any side effects of such chemicals) [46]. In each crop field and for each of the three sampling times, 50 plants were sampled over five random locations per field (10 plants per location), and we counted the number of adult and larval ladybeetles of each species and mixed aphid populations (i.e., *Rhopalosiphummaidis* (Fitch), *Macrosiphum miscanthi* (Takahashi), *Schizaphisgraminum* (Rondani), *R. padi* L.) on maize plants and *A. gossypii* on cotton plants through visual inspection of 50 plants in the fields [40]. For each species, all insects counted on the 50 plants were summed and doubled to obtain a single value per 100 plants per crop field and sampling date, and then the mean number of insects per 100 plants with the standard deviation was calculated for 83 study sites.

During each field survey, adult ladybeetles (at most 10 individuals per site) were collected from neighboring cotton and maize fields (300 m^2^ for each crop, at least 20 m away from survey locations to decrease the disturbance to the ladybeetle population abundance) to characterize their diet composition and host plant use, which were assessed through the measurement of their carbon isotope ratio. Only *P. japonica* (the most abundant species at the landscape scale, see results) was used. The collected individuals were placed into a 1.5 mL centrifuge tube containing 95% ethanol and stored at −20 °C for further stable isotope analysis following the method of Ouyang et al. [38] to assess the movement of this ladybeetle species between these two adjacent crops.

### 2.3. Statistical Analysis

#### 2.3.1. Field Scale

First, for each study year, we deployed a nonparametric test (Kruskal–Wallis test using “proc npar1way” of the SAS 9.4 software) to compare the difference in non-normality data of aphid density (AD) and ladybeetle metapopulation density (LMD)among these three plant taxonomic groups (crops, trees, weeds) across the whole sampling season. Then, we performed generalized linear mixed-effects models (GLMMs) to clarify the linear or nonlinear relationship between the response variable (LMD) and various explanatory variables [47], the model was fitted with negative binomial distributions due to the data overdispersionderived from the population fluctuation of aphids or ladybeetles on different plant species. The fixed effects included AD, a categorical variable of plant functional groups(Plant_FG with three levels: crops, trees, weeds) and their interaction, the sampling number of plant species (Plant_num) nested in each taxonomic group was the random effect. We first ran the full model which included all fixed effects, then, we removed nonsignificant effects by stepwise model selection, and the final model only contained one fixed effect of AD. Before the analysis, the abundance datawere log-transformed to decrease the model error estimated. GLMMs were fitted with the “glmer.nb” function in the “MASS” package [48] of the R 3.5.3 software [49].

#### 2.3.2. Regional Landscape Scale

At the regional landscape scale, we first ran a repeated-measures analysis with a mixed effects linear model (“proc mixed” within the SAS 9.4 software) to assess the variationin aphid density (AD) and ladybeetle metapopulation density (LMD) on cotton and maize in three investigative periods from June to August 2013. Month (with three levels: June, July, and August) was the repeated factor; crop (with two levels: cotton and maize), month and their interaction (crop*month) were the fixed effects; and the study site was the random effect. Then, in order to assess the response relationship between LMD and AD at the regional landscape scale, GLMM analysis with the Poisson distribution of non-overdispersion data using the R package “lme4” [50] was deployed. The fixed effects included AD, crop, study month, and the interactions of different explanatory variables, the study site was the random effect. Finally, because we were interested in how aphid density affected ladybeetle density, and the fixed effects were confirmed to be significant, all these main effects are shown in the tendency diagram. Prior to all analyses, the abundance data were transformed by log_10_(x + 1) to decrease the model error estimated.

Moreover, to study the temporal movement of ladybeetles between cotton and maize, stable carbon isotope analysis was used to detect the feeding activity of the dominating species, *P. japonica* adults, on aphids from cotton and/or maize fields, and the *δ*^13^ values were determined for the field-collected *P. japonica* samples (per crop and per sampling date). The criteria for estimating the proportion of aphidsin the diets of *P. japonica* adults that were from C_3_ or C_4_ plants (i.e., cotton or maize) followed the existing linear equation Y = 0.12X − 22.72, where Y is the *δ*^13^ value of *P. japonica* and X is the proportion of aphids from C_3_ plants and C_4_ plants [38].

At both field and landscape scales, we did a Chi-square test (“proc freq” within the SAS 9.4 software) to clarify the species composition proportion of aphidophagousladybeetles in different plant functional groups and crops, respectively.

## 3. Results

### 3.1. Species Composition of Aphidophagous Ladybeetles

There werethreeaphidophagous ladybeetle species (*C.septempunctata*, *P. japonica*, *H. axyridis*) found at both the local field and landscape scales (Figure S2). *H. axyridis* was the dominant ladybeetle specieson all plants at the local field scale, with proportions of 68.6% and 70.2% in 2013 and 2014, respectively; however, no significant difference was found for the species composition of aphidophagous ladybeetles in the different plant functional groups at the field scale in 2013 (χ^2^ = 0.65, df = 2, *p* = 0.723, Figure S2a) and 2014 (χ^2^ = 1.52, df = 2, *p* = 0.468, Figure S2b). More interesting, we found *P. japonica* was the dominant ladybeetle specieson these two main crops—cotton (97.6%) and maize (86.2%)—at the regional landscape scale in 2013, and significant differences were found for the species compositions of aphidophagous ladybeetles in different sampling months on cotton (χ^2^ = 8.96, df = 2, *p* = 0.011, Figure S2c) and maize (χ^2^ = 22.26, df = 2, *p* < 0.001, Figure S2d).

### 3.2. Effects of Aphids and Plant Taxa on Ladybeetles at the Field Scale

**Figure 1:**
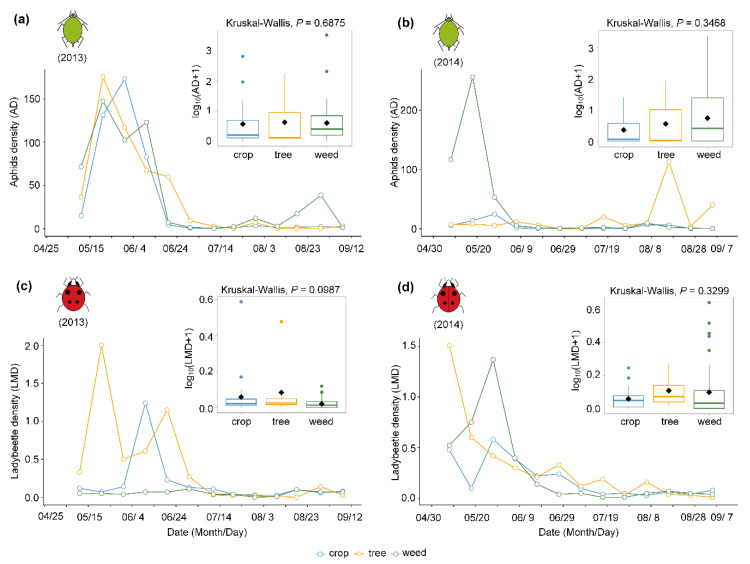
Population dynamics of aphids and ladybeetles on different plant functional groupsatthe field scale in 2013 and 2014. Aphid density (AD) in 2013 (**a**) and 2014 (**b**) and ladybeetle metapopulation density (LMD) in 2013 (**c**) and 2014 (**d**) are the mean values on each sampling date in three plant functional groups. Boxplots nested in the top right corner are the mean values of AD and LMD throughout the whole sampling period. Diamond points with black color in the box are the mean value for each functional plant taxon, whereas the line in each box is the median, and box edges represent the lower (Q1) and upper (Q3) quartiles. The number of plant species of plant functional groups (crops, trees, weeds) was 21, 8, and 49 species in 2013 and 26, 8, and 46 species in 2014, respectively. The *p* value indicated no significant difference (*p* > 0.05) between these plant taxa within the Kruskal–Wallis test.

At the field scale, both AD (aphid density) and LMD (ladybeetle metapopulation density) showed obvious seasonal dynamics from 2013 to 2014 (Figure 1). The ADs on three plant functional groups (i.e., crops, trees, and weeds) wereall higher from mid-May to early June in 2013 (Figure 1a), but the AD was only higheron weeds in mid-May in 2014 (Figure 1b). Meanwhile, the LMD on trees was higher in mid-May, and the peak value on crop plants was in early June in 2013 (Figure 1c), whereas the peak values on trees and weeds appeared in early May and late May in 2014, respectively(Figure 1d). However, there wereno significant differences inAD and LMD among the threeplant functional groups throughout the whole sampling period at the local field scale in 2013 (AD: χ^2^ = 0.75, df = 2, *p* = 0.688; LMD, χ^2^ = 4.63, df = 2, *p* = 0.099) and 2014 (AD: χ^2^ = 2.12, df = 2, *p* = 0.347; LMD, χ^2^ = 2.22, df = 2, *p* = 0.330) (small plotsnested in Figure 1), respectively.

**Figure 2:**
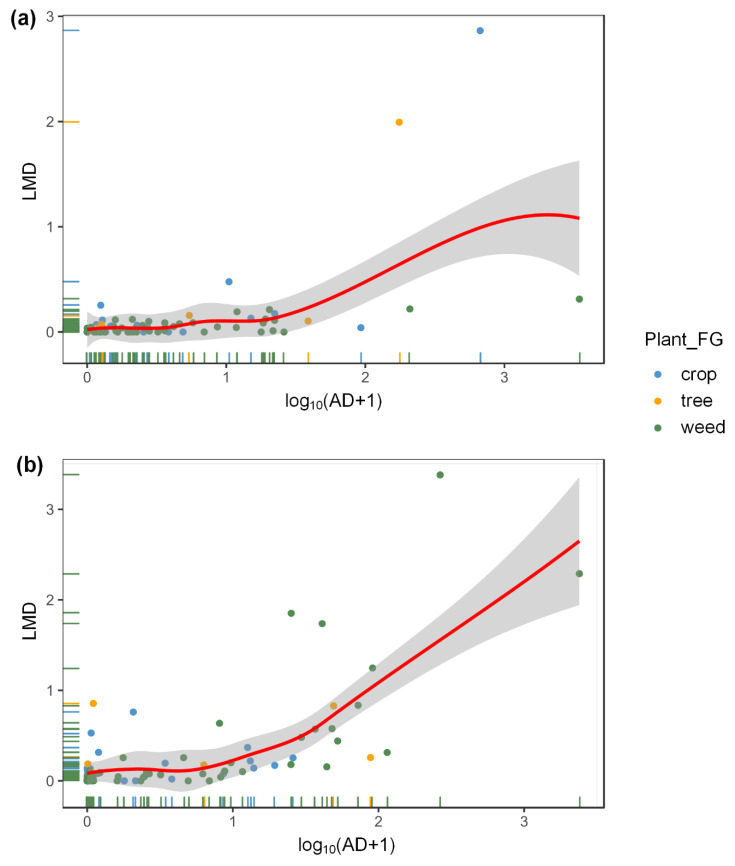
The relationship between aphid density (AD) and ladybeetle metapopulation density (LMD) atthe local field scale in 2013 and 2014. The statistical results of a negative binomial generalized linear mixed-effects model (GLMM model using the “glmer.nb” function in the “MASS” package of the R software) are shown in Table S2. Because theeffects of plant functional groups were not significant, we only provided the aphid effects here. The data points (with marginal rug) of different colors are for different plant functional groups, and the red smooth curve with 95% confidence interval band (shaded areas) is across all plant species in 2013 (**a**) and 2014 (**b**) due to no difference in population density in these functional plant taxa. Data on AD shown in the scatter plotswere transformed by log_10_(x + 1) before analysis.

For the GLMMs, no significant impacts were found for the interaction of aphid density (AD) and the three plant functional groups (Plant_FG) in 2013 (LRT chi-square test = 1.68, df = 2, *p* = 0.432) or 2014 (LRT chi-square test = 1.47, df = 2, *p* = 0.480) (AD: Plant_FG in Table S2, Figure S3a,b). However, the LMD wassignificantly positively related to AD across all plants in both years (2013: Wald Z = 4.14, *p* < 0.001, Figure 2a; 2014: Z = 5.62, *p* < 0.001, Figure 2b). These results indicated that aphid density was the main determinant of aphidophagouspredatory ladybeetle abundances at the local field scale.

Based upon the occurrence and distribution of plant species as well asaphid density on crops in northern China, the host plant shifts of aphidophagousladybeetles were determinedduring three successive periods atlocal farmland. From early May to early June, ladybeetles mainly remained on species including *Triticum aestivum* L., *Prunus persica* L., *Malus pumila* Mill., *Descurainiasophia* (L.) Webb. ex Prantl, *Hemisteptalyrata* Bunge, and *Populustomentosa* Carr. (seedling). From mid-June to early August *Gossypium hirsutum* L., *Vitis vinifera* L., *Artemisia lavandulaefolia* DC., *A. annua* L., *P. tomentosa* (seedling), etc., were the dominant habitat plants of ladybeetles. In the last periods, i.e., from mid-August to early September, a large number of predatory ladybeetles moved to *Zea mays* L., *A. lavandulaefolia*, *Chenopodium album* L., *Ulmuspumila* L., etc. (Figure S4).

### 3.3. Effects of Aphids on Ladybeetles at the Regional Landscape Scale

**Figure 3:**
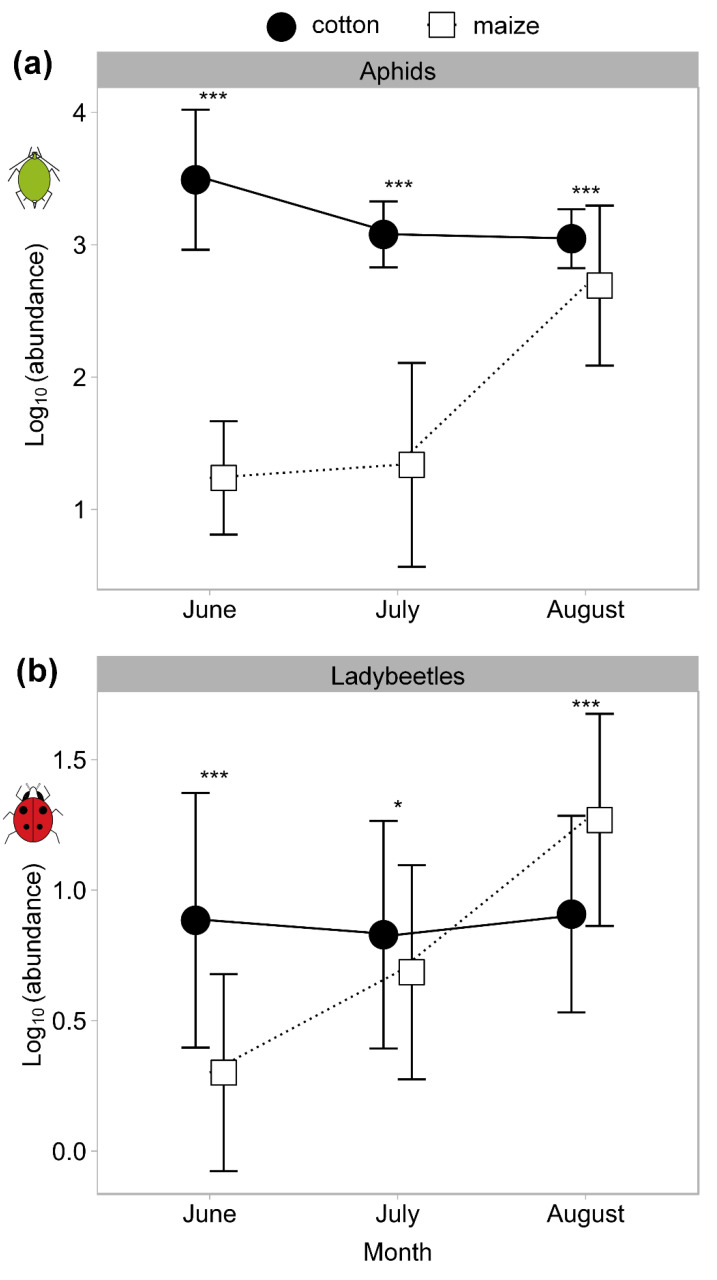
The population density of aphids and ladybeetles in different sampling periods (months) on cotton and maize crops at the regional landscape scale in 2013. (**a**): aphid density (AD); (**b**): ladybeetle metapopulation density (LMD). The data are the average ± S.E. with log_10_(x + 1) transformed before using repeated-measures analysis with a mixed effect linear model (“proc mixed” in the SAS software). Asterisk (*) indicates the significant difference between cotton and maize in different sampling months, * *p* < 0.05, *** *p* < 0.001.

At the regional landscape scale, the AD varied throughout the months (*F* = 81.48, df = 2, 328, *p* < 0.001) on both cotton and maize and was higher on cotton than maize throughout the whole sampling period (*F* = 941.03, df = 1, 82, *p* < 0.001). The AD on cotton decreased from June to July and August but that on maize increased from June and July to August (Figure 3a). The mean LMDabundance was higher on cotton than on maize throughout the whole sampling period (*F* = 10.97, df = 1, 82, *p* = 0.001). The LMD on maizewas also higher in August than in June (*t* = 16.18, *p* < 0.001) and July (*t* = 10.2, *p* < 0.001), but we did not find a significant decrease in the LMD on cotton (August vs June: *t* = 0.39, *p* = 0.694; August vs July: *t* = 1.38, *p* = 0.168) (Figure 3b). There were also significant differences in the AD or LMD between cotton and maize in each sampling month (AD, June: *t* = 28.77, *p* < 0.001; July: *t* = 22.24, *p* < 0.001; August: *t* = 4.52, *p* < 0.001. LMD, June: *t* = 9.72, *p* < 0.001; July: *t* = 2.39, *p* = 0.017; August: *t* = −6.00, *p* < 0.001). Moreover, LMD was enhanced following the increasing AD on maize, which indicated a following relationship between the ladybeetle andaphid population.

**Figure 4:**
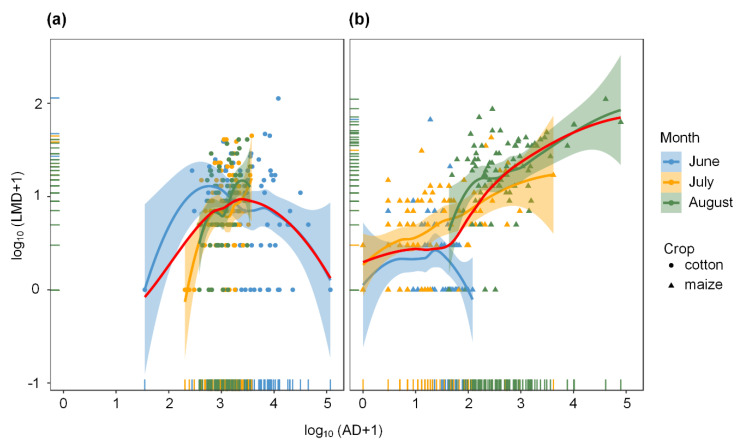
The relationship between aphid density (AD) and ladybeetle metapopulation density (LMD) in different sampling periods (months) on cotton and maize crops at the regional landscape scale in 2013. The data are transformed by log_10_(x + 1) before using GLMM analysis with Poisson distribution (“lme4” package of the R software). The scatter plot with different colors shows the data points (with marginal rug) and the regression trend lines (smooth curves) and 95% confidence interval band (shaded areas) in different months (June: steel blue color; July: orange color; August: pale green color). The smooth regression curve in red indicates the trend across the entire sampling period (from June to August). Circle points and triangle points indicate the data from cotton (**a**) and maize (**b**), respectively. The statistical results of GLMMs are shown in Table 1.

Furthermore, the GLMM analysis also revealed the relationship between the occurrence of ladybeetles and aphids at the regional landscape scale (Table 1). LMD was significantly positively related to AD (Wald Z = 3.93, *p* < 0.001), and the interactions such as AD:crop (LRT chi-square test = 100.18, df = 1, *p* < 0.001) and AD:month (LRT chi-square test = 223.38, df = 2, *p* < 0.001) were both significant, which indicated the positive effects of AD on LMD were also significant between different crops and different sampling periods. For individual crop, the positive effect of the AD on LMD was only significant on maize (Z = 91.37, *p* < 0.001, AD:crop-maize interaction) rather than cotton among different sampling periods (from June to August) (Figure 4). The positive relationship between LMD and AD was also significant in July (Z = 6.01, *p* < 0.001, AD:month-July interaction) and August (Z = −87.56, *p* < 0.001, AD:month-August interaction) on both cotton (Figure 4a) and maize (Figure 4b).

In the stable carbon isotope analysis, the *δ*^13^Cvalues of the *P. japonica* adults from the cotton and maize fields showed that they were nearly all positive (~100%) for consumption of aphids originated from C_3_plants in June and July. In August, ladybeetles in cotton and maize are mostly resident in the crop but there is a slight migration between the two crops, i.e., 13.43% of ladybeetles in cotton came from maize (C_4_plants) and 28.29% in maize came from cotton (C_3_plants) (Table 2). These results indicated thatmost prey aphids for ladybeetles originated from crops where aphids areabundant (cotton in June and July; both maize and cotton in August), and ladybeetle density had a positive response to aphid density on these two crops following their growing period.

## 4. Discussion

Prey densities can affect the host plant selection and population growth of predatory ladybeetles [1,8]. In our survey, we provided further evidence that aphid densities are a strong determinant of ladybeetledensities in the farmlands of northern China, and that the host plant type also affected ladybeetle densities at a regional landscape scale. Especially at the local field scale, the trees hosted high ladybeetle densities in the middle of spring, while the crops hosted high ladybeetle densities at the end of spring. The stable carbon isotope analysis at the regional landscape survey confirmed that ladybeetles moved between crops following aphid population fluctuations.

In our two progressive surveys (local field scale and regional landscape scale), the ladybeetle densities increased with increasing aphid densities, as expected and consistent with earlier literatures [9,51,52,53,54]. The ladybeetles responded differently depending on the host plant type: they responded to high aphid densities in maize fields but had a less obvious response to those in cotton fields at the regional landscape scale, which was similar to the results of Ouyang et al. [38], and this response possibly occurred through a phenology effect/host shift, which is discussed below. At the local field scale in 2013, the ladybeetles responded more strongly to aphid densities in trees and crops than weeds. This may be due to the difference in the compensation of plant functional diversity [55]. In addition to prey, other plant nutrients can also benefit predators. For instance, Bertrand et al. [56] reported that two generalist predators of crop aphids (a ladybeetle species: *H. axyridis*, and a lacewing species: *Chrysoperlacarnea*) could utilize pollen sources from trees (e.g., *Salix*, *Prunus*, *Quercus*, and *Acer*) to survive during seasonal shifts in resources in agricultural landscapes. Plant-provided food supplements can affect diversity and biological pest control by omnivorous predators [57]. Some non-crop plants could attract and conserve aphid predators in specific crop fields [58], and plant morphology may affect ladybeetle mobility, foraging behavior, and access to prey, encouraging them to forage on preferred plant types [59,60]. For instance, Reynolds and Cuddington [61] showed that *H. axyridis* foraged more thoroughly and had a higher foraging ability on plants with more branches compared with leafier plants. *H. axyridis* also preferred to aggregate in the more sunny section of tree crowns [62], and they were of a higher abundance on trees than on herbaceous plants and cereals in central Europe [63,64]. Hence, trees would be a preferred habitat for them. Different plant species attacked by aphids may also be differentially attractive to ladybeetles due to differences in susceptibility to herbivorous attacks and in the emission of herbivore-induced plant volatiles [65,66,67,68,69]. Finally, the area occupied by the focal plant species in the local landscape may also affect ladybeetles’ foraging behavior via a concentration effect, i.e., plant species occupying large areas such as crops and trees may represent large, uniform, and easily detected prey reservoirs and hence be preferred by ladybeetles [70,71].In addition, a more complex landscape not only can increase plant functional diversity, but can also create the microclimate to influence the thermal tolerance of aphid, beetles, and parasitoids, which in turn could impact biological control strategies [72,73,74]. As the global climate warms, insectpests and their biological control agents maybe a mismatch, as suggested by Tougeron et al. [73] on aphid parasitoids, landscape complexity however can to some extent alleviate the influence of climatechange on the interaction of prey–predator.

In the regional landscape-scale survey, the stable carbon isotope analysis showed that adult ladybeetles moved from aphid-depleted patches to patches with high aphid density: the *P. japonica* adults collected in maize fields in June and July mostly preyed upon aphids originating from C_3_plants (likely cotton, the most abundant C_3_ plant in the environment). This suggested that they hunted for prey in the fields with the highest aphid densities, i.e., cotton fields in June and July. In August, the percentage of aphids consumed by *P. japonica* adults that had developed on C_4_plants (likely maize, the most abundant C_4_ plant in the environment) increased when compared with June and July. This result was consistent with the population growth of aphids in maize fields and the concomitant decrease in aphid densities in cotton fields from July to August. Similar prey-motivated changes in habitat selection by ladybeetles tracking high aphid densities have been observed for other ladybeetle species [1,21,75]. Additionally, crop phenology or temperature shifts can cause the population movement of natural enemies, for instance, maizeplants at whorl stage offer suitable micro-climatic conditions forforaging ladybeetlesand could act as a refuge during seasonally hot or dry conditions in agro-ecosystems of northern China [76]. Thus, global landscape management such as increasing plant diversification could improve conservation biological control in agro-ecosystems [77,78].

Evidence for temporal host plant shifts was also found at the local field scale, with ladybeetle densities decreasing in trees over time in 2013 and 2014 and increasing versus decreasing in weeds over time in 2013 and 2014, respectively, compared with in crops. Hence, trees may represent early season reservoirs forladybeetles before colonization of crops or weed patches [62]. However, the aphid densities in crops were already high in early May when many ladybeetles were in trees, which also supported high aphid densities; therefore, their shift from trees to crops may happen too late for efficient biocontrol of aphid populations in crop field [79,80]. There were also likely temporal shifts within plant types (e.g., from cotton to maize) that could not be tested with our models. Seasonal migrations of ladybeetles across host plants have often been described and proposed as a means to enhance aphid biocontrol via habitat enhancement and the conservation of reservoirs [3,8].

Interspecific competition between ladybeetle species determines their foraging behavior, especially in patch selection for oviposition [53,81,82,83]. In the present study, ladybeetle densities were significantly positively related toaphid densities at the local field and landscapescales, hinting at the absence of differential specialization to aphid densitiesby different ladybeetle species. However, we found that *H. axyridis* was the dominant ladybeetle species at the local field scale, whereas *P. japonica* was the dominant ladybeetle species at the landscape scale. This may be because we only surveyed two crops (i.e., cotton and maize) at the landscape scale, while there are 96 common plant species(crops, trees, and weeds) at the local field scale, which increase the diversity of host plant of their prey, especially *H. axyridis* more preferred to settle on trees compared with crop plants. Meanwhile, *P. japonica* maybe more competitive and more rapidly builds populations on cotton and maize crops, although *H. axyridis* has been found to be a strong intraguild competitor [2,84]. In Chinese agroecosystems, *H. axyridis* has greater predatory capacity on eggs of *C. septempunctata* and *P. japonica* in laboratory choice and no-choice trials, and all predator–prey combinations in the field yieldedpositive documentation of intraguild predation by using DNA-based gut-content detection [85]. Additionally, the two species may have slightly shifted phenologies [86], allowing decreased interspecific competition between them.

## 5. Conclusions

In summary, we reported that aphid density was a key determinant of predatory ladybeetle abundances, and plant taxa also to a certain extent affected ladybeetle densities. Host plant species likely influenced ladybeetle foraging behavior through temporal host shifts. Hence, it would be helpful to better characterize host plant reservoirs and temporal host shifts in more complex landscapes, as well as the spatial cues related to food resources, since non-crop habitats are also heavily used by predatory ladybeetles. By improving non-crop and crop habitats in synchrony with crop development and aphid population growth, it would help to maximize predatory ladybeetle movements from non-crop plants into crop fields, there by promote conservation biocontrol of aphid populations by predatory ladybeetles.

## Figures and Tables

**Table 1:** Generalized linear mixed models (GLMMs) related ladybeetle metapopulation density (LMD) with aphid density (AD) on cotton and maize crops in different months at the regional landscape scale.The fixed effects included AD, crop, month, and interactions of different explanatory variables. Estimates and S.E. was the coefficient and standard error of the fixed effect. The bold *p* value indicates a significant effect (*p* < 0.05).

Fixed Effects	Estimates	S.E.	Wald Z	*p*
(Intercept)	1.85600	0.070920	26.17	<0.001
AD	0.00001	0.000003	3.93	< 0.001
Crop_maize	−0.16050	0.029880	−5.37	<0.001
Month_August	1.06200	0.035830	29.65	<0.001
AD:crop_maize	0.00035	0.000004	91.37	<0.001
AD:month_July	0.00018	0.000031	6.01	<0.001
AD:month_August	−0.00034	0.000004	−87.56	<0.001

**Table 2:** Estimated proportion of diet for *Propylea japonica* adults originating from C_3_ (cotton) and C_4_ (maize) plants at a regional landscape composed of cotton and maize in 2013. The carbon isotope ratio*δ*^13^Cdata are shown as the mean ±SE.

Crops	June				July				August			
	No. Samples	Carbon Isotope Ratio*δ*^13^C	C_3_ Plant	C_4_ Plant	No. Samples	Carbon Isotope Ratio*δ*^13^c	C_3_ Plant	C_4_ Plant	No. Samples	Carbon Isotope Ratio*δ*^13^c	C_3_ Plant	C_4_ Plant
Cotton	119	−26.70 ± 1.01	~100.00%(119)	~0.00%(0)	101	−25.46 ± 1.78	~100.00%(101)	~0.00%(0)	458	−21.11 ± 5.92	~86.57%(396)	~13.43%(62)
Maize	199	−26.62 ± 1.51	~100.00%(199)	~0.00%(0)	141	−23.72 ± 3.91	~100.00%(141)	~0.00%(0)	852	−14.12 ± 5.01	~28.29%(241)	~71.71%(611)

## Supplementary Materials

The following are available online at https://www.mdpi.com/2075-4450/11/10/695/s1. Additional Supplementary Information may be found in the online version of this article: Table S1: Plant species and plant functional groups surveyed at the local field scale in 2013 and 2014. Table S2: Effects of aphids and plant taxa on ladybeetle metapopulation density (LMD) at the local field scale. Figure S1: The distribution of 83 study sites at the regional landscape scale in northern China in 2013. Figure S2: The species composition of aphidophagous ladybeetles at the local field and landscape scales. Figure S3: The GLMM analysis results for the effects of aphid density on ladybeetle metapopulation density in different plant functional groups at the local field scale. Figure S4: Dominant host plant species of ladybeetles and corresponding aphid densities at the local farmland scale from the surveys in 2013 and 2014.
