# Supplementary material for: Effects of Aphid Density and Plant Taxa on Predatory Ladybeetle Abundance at Field and Landscape Scales
Source: Insects. 2020 Oct 13;11(10):695. doi: 10.3390/insects11100695 (PMC7602106; doi:10.3390/insects11100695)
Supplement: Supplementary file 1 [file insects-11-00695-s001.pdf]

Effects of Aphid Density and Plant Taxa on Predatory Ladybeetle Abundance at Field and Landscape Scales

Supplementary Materials

Contents

Table S1: Plant species and plant functional groups surveyed at the local field scale in 2013 and 2014.

Table S2: Effects of aphids and plant taxa on ladybeetle metapopulation density (LMD) at the local field scale.

Figure S1: The distribution of 83 study sites at the regional landscape scale in northern China in 2013.

Figure S2: The species composition of aphidophagous ladybeetles at the local field and landscape scales.

Figure S3: The GLMM analysis results for the effects of aphid density on ladybeetle metapopulation density in different plant functional groups at the local field scale.

Figure S4: Dominant host plant species of ladybeetles and corresponding aphid densities at the local farmland scale from the surveys in 2013 and 2014.

Table S1. Plant species and plant functional groups surveyed at the local field scale in 2013 and 2014. A total of 96 plant species were recorded in these two years, and all plant species were divided into three plant taxa (31, 12, and 53 species of crops, trees, and weeds, respectively) based on the vegetation of the habitats.

Family	Plant species	2013	2014	Plant_taxa
Amaranthaceae	*Amaranthus tricolor* L.	+	+	weed
Asclepiadaceae	*Metaplexis japonica* (Thunb.) Mak.	+	+	weed
Asteraceae	*Artemisia annua* L.	+	+	weed
Asteraceae	*Artemisia lavandulaefolia* DC.	+	+	weed
Asteraceae	*Artemisia scoparia* Waldst. et Kit.	+	+	weed
Asteraceae	*Calendula officinalis* L.	+		weed
Asteraceae	*Cephalanoplos setosum* (Willd.) Kitam.	+	+	weed
Asteraceae	*Cirsium setosum* (Willd.) MB.	+	+	weed
Asteraceae	*Conyza canadensis* (L.) Cronq.	+	+	weed
Asteraceae	*Coreopsis tinctoria* Nutt.	+		weed
Asteraceae	*Helianthus annuus* L.	+	+	crop
Asteraceae	*Helianthus tuberosus* L.	+		crop
Asteraceae	*Hemistepta lyrata* Bunge	+	+	weed
Asteraceae	*Heteropappus altaicus* (Willd.) Novopokr.	+	+	weed
Asteraceae	*Ixeris chinensis* (Thunb.) Nakai.	+		weed
Asteraceae	*Ixeris denticulata* (Houtt.) Stebb.	+	+	weed
Asteraceae	*Ixeris sonchifolia* Hance	+	+	weed
Asteraceae	*Sonchus brachyotus* DC.	+	+	weed
Asteraceae	*Xanthium sibiricum* Patrin ex Widder	+	+	weed
Brassicaceae	*Brassica oleracea* L.		+	crop
Brassicaceae	*Descurainia sophia* (L.) Webb. ex Prantl	+	+	weed
Brassicaceae	*Lepidium apetalum* Willd.	+	+	weed
Brassicaceae	*Orychophrapmus violaceus* (L.) O. E. Schulz	+	+	weed
Chenopodiaceae	*Chenopodium album* L.	+	+	weed
Chenopodiaceae	*Chenopodium glaucum* L.	+	+	weed
Chenopodiaceae	*Chenopodium serotinum* L.	+	+	weed
Chenopodiaceae	*Kochia scoparia* (L.) Schrad.	+	+	weed
Chenopodiaceae	*Salsola collina* Pall.	+	+	weed
Convolvulaceae	*Calystegia dahurica* (Herb.) Choisy	+	+	weed
Convolvulaceae	*Calystegia hederacea* Wall.	+		weed
Convolvulaceae	*Convolvulus arvensis* L.	+	+	weed
Convolvulaceae	*Pharbitis nil* (L.) Choisy	+	+	weed
Convolvulaceae	*Pharbitis purpurea* (L.) Voight	+	+	weed
Cucurbitaceae	*Cucurbita moschata* (Duch.) Poiret		+	crop
Equisetaceae	*Equisetum ramosissimum* Desf.	+		weed
Euphorbiaceae	*Acalypha australis* L.	+	+	weed
Euphorbiaceae	*Euphorbia helioscopia* L.		+	weed

Fabaceae	*Amorpha fruticosa* L.		+	tree
Fabaceae	*Arachis hypogaea* L.	+	+	crop
Fabaceae	*Glycine max* (L.) Merr.	+	+	crop
Fabaceae	*Medicago falcata* L.	+	+	weed
Fabaceae	*Pisum sativum* L.	+		crop
Fabaceae	*Robinia pseudoacacia* L.		+	tree
Fabaceae	*Sophora japonica* L.	+		tree
Fabaceae	*Sophora japonica* L. var. japonica f. pendula		+	tree
Fabaceae	*Vigna angularis* (Willd.) Ohwi et Ohashi		+	crop
Fabaceae	*Vigna radiata* (L.) Wilczek	+		crop
Fabaceae	*Vigna unguiculata* (L.) Walp.		+	crop
Lamiaceae	*Lagopsis supina* (Steph.) IK.-Gal.	+	+	weed
Lamiaceae	*Leonurus heterophyllus* Sweet	+		weed
Lamiaceae	*Mentha haplocalyx* Briq.		+	weed
Lamiaceae	*Salvia plebeia* R. Br.	+		weed
Liliaceae	*Allium fistulosum* L.	+		crop
Liliaceae	*Allium tuberosum* Rottl. ex Spreng.		+	crop
Malvaceae	*Abutilon theophrasti* Medic.	+	+	weed
Malvaceae	*Gossypium hirsutum* L.	+	+	crop
Moraceae	*Cannabis sativa* L.	+	+	crop
Moraceae	*Humulus scandens* (Lour.) Merr.	+	+	weed
Moraceae	*Morus alba* L.	+	+	tree
Pedaliaceae	*Sesamum indicum* L.	+		crop
Pinaceae	*Pinus tabulaeformis* Carr.	+		tree
Plantaginaceae	*Plantago depressa* Willd.	+	+	weed
Poaceae	*Eleusine indica* (L.) Gaertn.		+	weed
Poaceae	*Phragmites communis* Trin.	+	+	weed
Poaceae	*Setaria viridis* (L.) Beauv.	+	+	weed
Poaceae	*Sorghum vulgare* Pers.		+	crop
Poaceae	*Triticum aestivum* L.	+	+	crop
Poaceae	*Zea mays* L.	+	+	crop
Polygonaceae	*Polygonum aviculare* L.		+	weed
Portulacaceae	*Portulaca oleracea* L.	+	+	weed
Rhamnaceae	*Ziziphus jujuba* Mill.	+	+	crop
Rosaceae	*Crataegus pinnatifida* Bge.	+	+	crop
Rosaceae	*Malus pumila* Mill.	+	+	crop
Rosaceae	*Potentilla supina* L.	+	+	weed
Rosaceae	*Prunus armeniaca* L.	+	+	crop
Rosaceae	*Prunus cerasifera* Ehrh.	+	+	crop
Rosaceae	*Prunus persica* L.	+	+	crop
Rosaceae	*Prunus salicina* Lindl.	+	+	crop
Rosaceae	*Pyrus bretschneideri* Rehd.	+	+	crop

Rubiaceae	*Rubia cordifolia* L.	+	+	weed
Rutaceae	*Zanthoxylum bungeanum* Maxim.		+	crop
Salicaceae	*Populus tomentosa* Carr.	+	+	tree
Salicaceae	*Salix matsudana* Koidz.	+	+	tree
Scrophulariaceae	*Paulownia fortunei* (Seem.) Hemsl.	+		tree
Scrophulariaceae	*Rehmannia glutinosa* Libosch.	+	+	weed
Simaroubaceae	*Ailanthus altissima* (Mill.) Swingle	+		tree
Solanaceae	*Capsicum annuum* L.		+	crop
Solanaceae	*Lycium chinense* Miller	+	+	weed
Solanaceae	*Lycopersicon esculentum* Mill.		+	crop
Solanaceae	*Solanum melongena* L.		+	crop
Solanaceae	*Solanum nigrum* L.	+	+	weed
Sterculiaceae	*Firmiana platanifolia* (L. f.) Marsili		+	tree
Ulmaceae	*Ulmus pumila* L.	+	+	tree
Violaceae	*Viola philippica* Cav.	+	+	weed
Vitaceae	*Vitis vinifera* L.	+	+	crop
Zygophyllaceae	*Tribulus terrester* L.	+	+	weed

Note: + indicates that this plant species was surveyed in the corresponding year. A blank space indicates that the plant was not surveyed in that year.

Table S2. Effects of aphids and plant taxa on ladybeetle metapopulation density (LMD) at the local field scale. Generalized linear mixed-effects models (GLMMs) with a negative binomial distribution (function of "glmer.nb" in the "MASS" package of R) were used to analyze the data. The fixed effects included aphid density (AD), plant functional groups (Plant_FG) and their interactions (AD:Plant_FG). Plant_num was the random effect. Estimates and S.E. was the coefficient and standard error of the fixed effect. The bold *P* value indicates a significant effect ($P < 0.05$). The LRT (likelihood ratio test) chi-square test explained the interaction.

Model selection	Year	Fixed effects	Estimates	S.E.	Wald Z	*P*	R^2_m	R^2_c	AIC
Full model	2013	(Intercept)	-3.41	0.98	-3.50	**0.0005**	0.226	0.226	51.1
		AD	1.52	0.44	3.48	**0.0005**			
		Plant_FGtree	-0.44	2.13	-0.21	0.8365			
		Plant_FGweed	-0.29	1.33	-0.22	0.8271			
		AD:Plant_FGtree	0.44	1.04	0.43	0.6712			
		AD:Plant_FGweed	-0.73	0.69	-1.06	0.2902			
	2014	(Intercept)	-2.07	0.60	-3.46	**0.0005**	0.267	0.267	93.0
		AD	0.46	0.80	0.57	0.5696			
		Plant_FGtree	0.63	1.03	0.61	0.5431			
		Plant_FGweed	-0.48	0.76	-0.64	0.5252			
		AD:Plant_FGtree	-0.05	1.06	-0.05	0.9593			
		AD:Plant_FGweed	0.73	0.83	0.89	0.3760			
Final model	2013	(Intercept)	-3.48	0.67	-5.19	**2.1E-07**	0.191	0.213	50.7
		AD	1.21	0.29	4.14	**3.6E-05**			
	2014	(Intercept)	-2.37	0.37	-6.47	**9.8E-11**	0.284	0.284	86.6
		AD	1.10	0.20	5.62	**2.0E-08**			

The interaction effect of the AD and Plant_FG of the full model:

Year	Fixed effects	df	AIC	LRT	Pr (chi)
2013	AD:Plant_FG	2	48.77	1.68	0.4315
2014	AD:Plant_FG	2	90.48	1.47	0.4801

§: R^2_m is the marginal R^2, which represents the variance explained only by the fixed effects, whereas R^2_c is the conditional R^2, which represents the variance explained by both fixed and random effects. The full model is "LMD ~ AD + Plant_FG + AD:Plant_FG + (1|Plant_num)". The final model is "LMD ~ AD + (1|Plant_num)" after removing the nonsignificant effects of Plant_FG and the interactions (AD:Plant_FG).

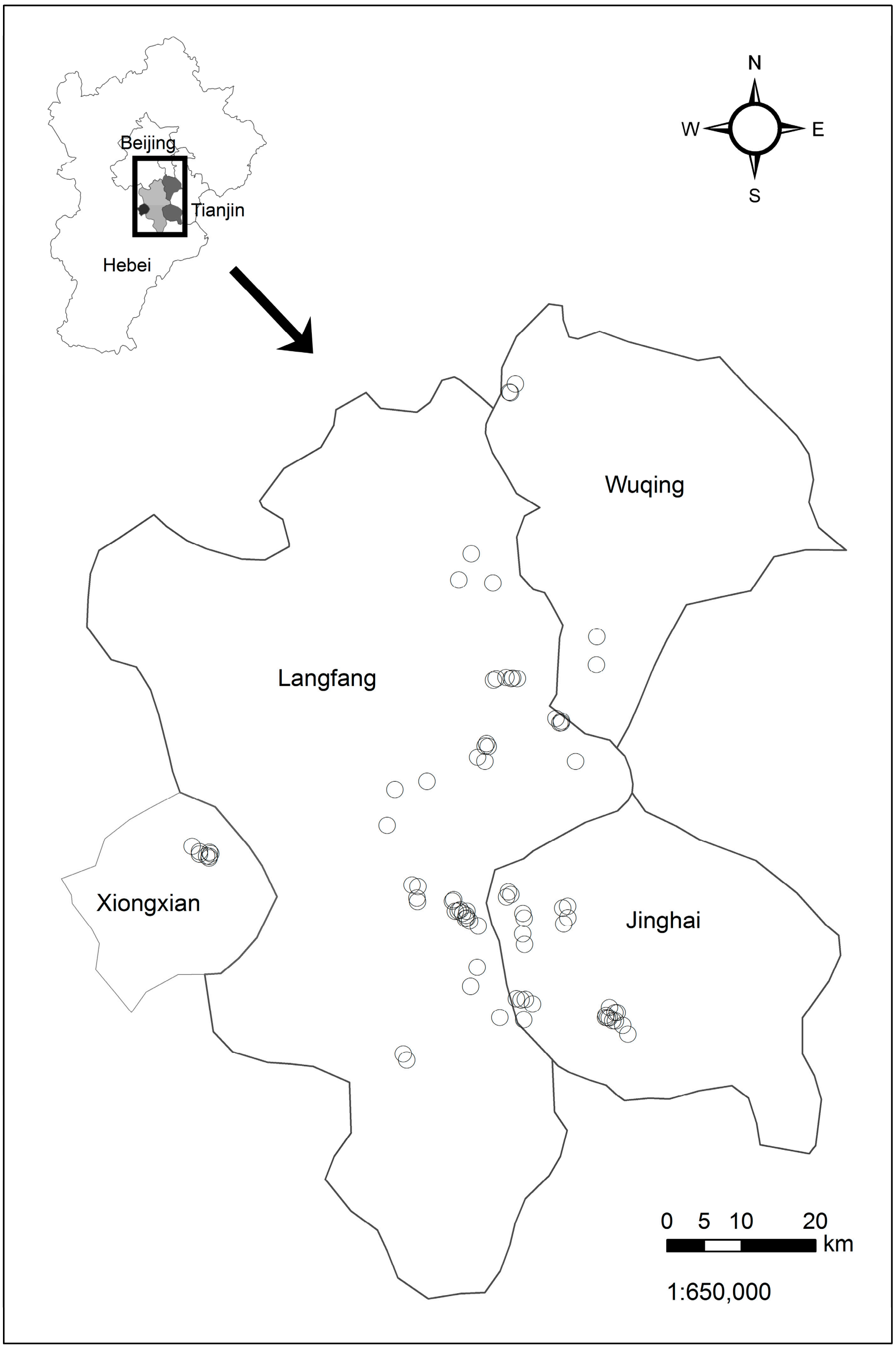

Figure S1. The distribution of 83 study sites at the regional landscape scale in northern China in 2013. The 83 study sites spanned four cities: Langfang city and Xiongxian County (belonging to Baoding city) in Hebei Province and Wuqing and Jinghai in Tianjin Province. Each circle indicates one study site.

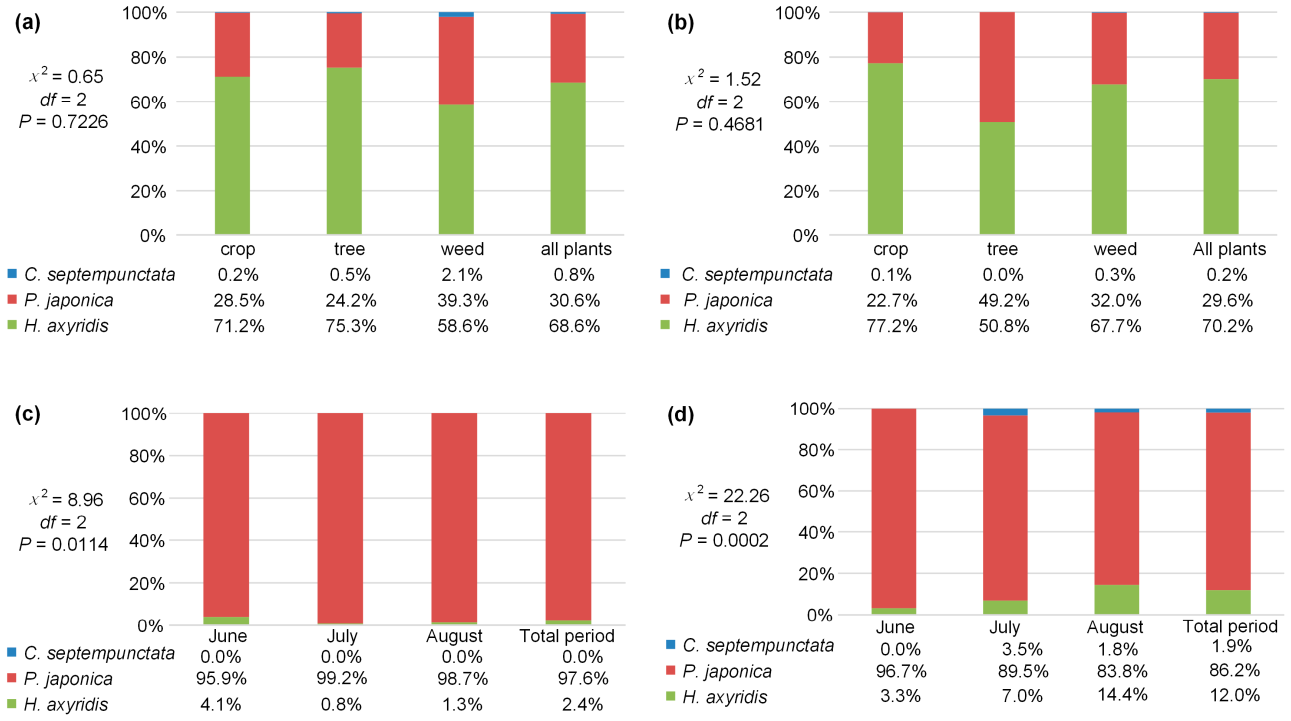

Figure S2. The species composition of aphidophagous ladybeetles at the local field and landscape scales. The proportion of aphidophagous ladybeetles in different plant functional groups at the local field scale in 2013 (**a**) and 2014 (**b**) and cotton (**c**) and maize (**d**) in different months at the regional landscape scale in 2013. The data below the bars represent the proportion of each ladybeetle species. The chi-squared test shows the significance of the species composition.

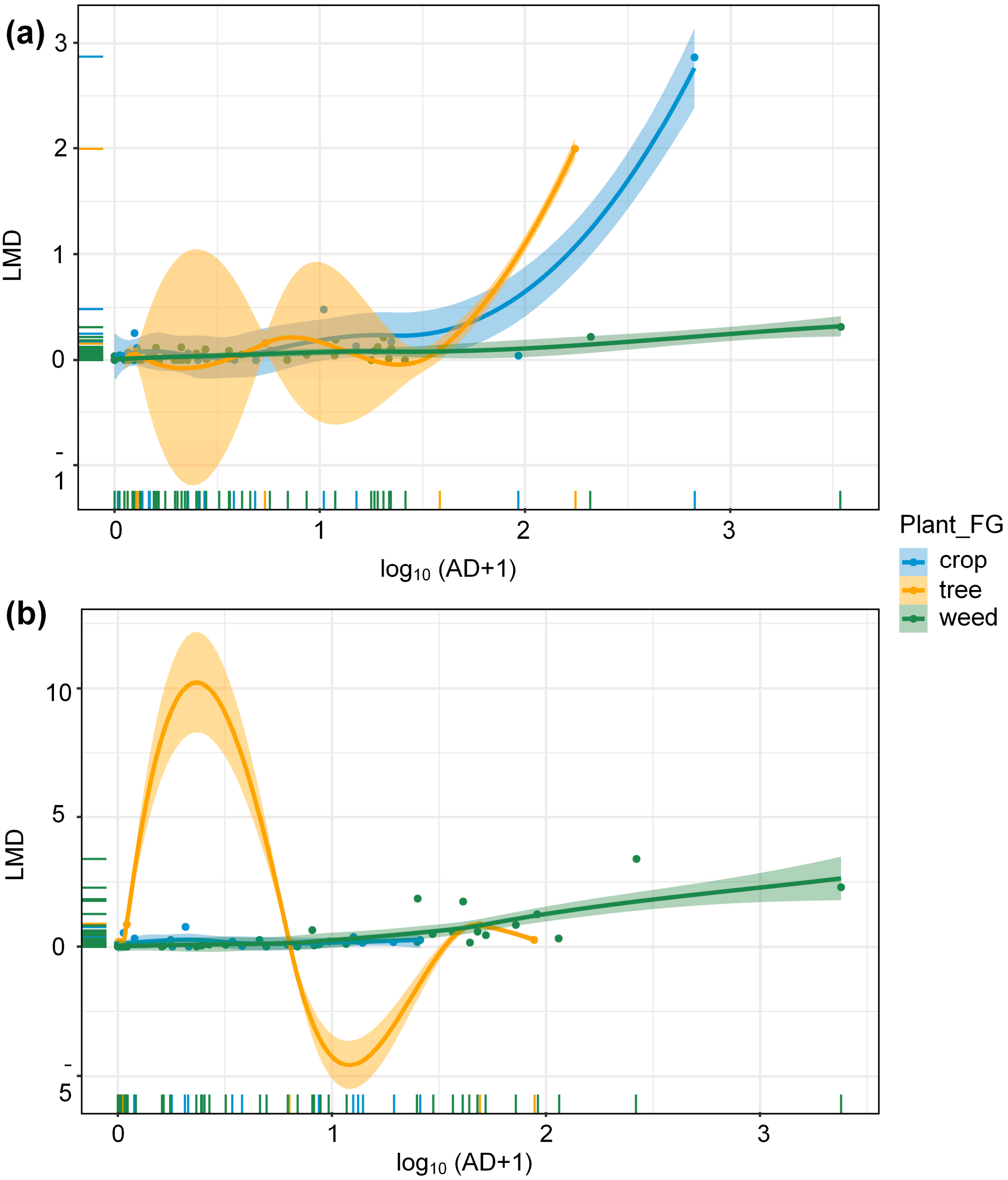

Figure S3. The GLMM analysis (primary model) results for the effects of aphid density (AD) on ladybeetle metapopulation density (LMD) in different plant functional groups at the local field scale. The GLMM model, which was created using the "glmer.nb" function in the "MASS" package of R software, is shown in Table S2. The data points and marginal rug and the smooth curve with 95% confidence interval bands (shaded areas) of different colors show the different plant functional groups in 2013 (**a**) and 2014 (**b**). The data on AD shown in the scatter plots were transformed by $\log_{10}(x+1)$ before analysis.

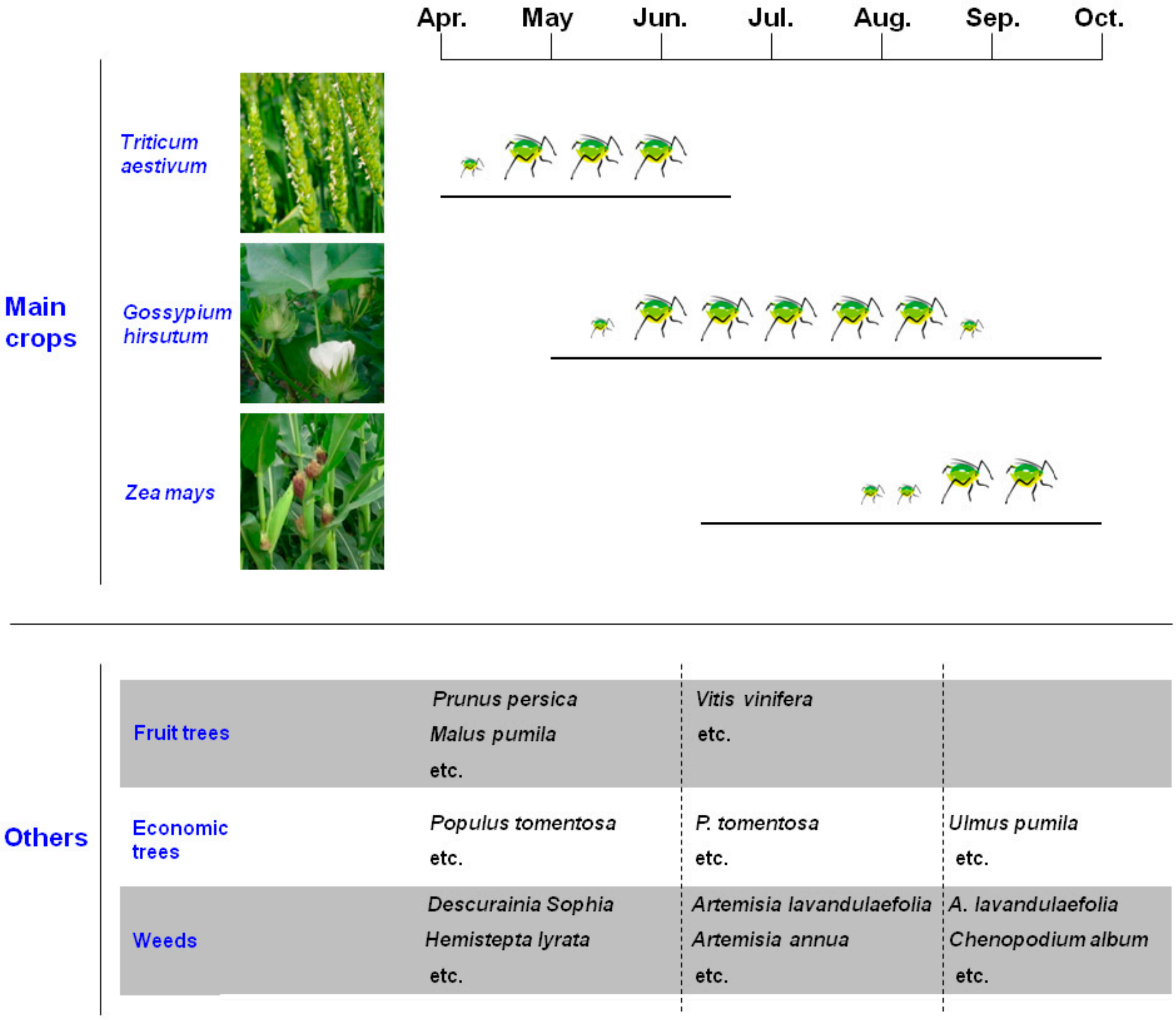

Figure S4. Dominant host plant species of ladybeetles and the corresponding aphid densities at the local farmland scale from the surveys in 2013 and 2014. The sizes of the aphid images indicate the population abundance of aphids on each main crop.
